# Mucosa-associated lymphoid tissue lymphoma of the trachea associated with idiopathic pulmonary fibrosis: A case report and literature review

**DOI:** 10.1097/MD.0000000000010727

**Published:** 2018-05-18

**Authors:** June Hong Ahn, Jin Hong Chung, Kyeong-Cheol Shin, Eun Young Choi, Hyun Jung Jin, Joon Hyuk Choi, Kwan Ho Lee

**Affiliations:** a Department of Internal Medicine; b Department of Pathology, Regional Center for Respiratory Disease, Yeungnam University Medical Center, College of Medicine, Yeungnam University, Daegu, Republic of Korea.

**Keywords:** B-cell, idiopathic pulmonary fibrosis, lymphoma, marginal zone, trachea

## Abstract

**Rationale:**

Mucosa-associated lymphoid tissue (MALT) lymphoma of the trachea is a rare disease that has been shown to be associated with chronic antigenic stimulation. There have been few reports of MALT lymphoma of the trachea in association with idiopathic pulmonary fibrosis (IPF).

**Patient concerns:**

A 73-year-old patient visited with a 2-year history of dyspnea on exertion and productive cough, which had worsened 1 month ago.

**Diagnoses:**

MALT lymphoma of the trachea associated with IPF.

**Interventions:**

After taking into consideration the age, poor performance status, and comorbidities of the patient and the extent of disease, we utilized an observational approach as a treatment strategy.

**Outcomes:**

The patient is well without any evidence of progression for 12 months since the initial diagnosis.

**Lessons:**

We present a case of MALT lymphoma of the trachea associated with IPF. A common predisposing factor may exist for tracheal MALT lymphoma and IPF. As there are no randomized clinical trials focusing on tracheal MALT lymphoma, individualized treatment decision is important, and in some cases, simply monitoring the patient might be the most appropriate approach.

## 1 Introduction

Primary pulmonary lymphoma (PPL) is a rare disease that is defined as a clonal lymphoid proliferation arising from the lung. Mucosa-associated lymphoid tissue (MALT) lymphoma is the most common type of indolent B-cell PPL, originating from postgerminal center memory B-cells. MALT lymphomas are generally associated with chronic antigenic stimulation in response to cytogenetic abnormalities, autoimmune disease, or chronic pulmonary infection.^[1]^ MALT lymphomas arising in the tracheal wall are extremely rare, and fewer than 20 cases of primary tracheal MALT lymphomas have been reported in the literature.^[2]^

Idiopathic pulmonary fibrosis (IPF) is the most common form of idiopathic interstitial pneumonia. IPF has been suggested to occur in genetically vulnerable individuals as a consequence of an excessive wound healing reaction following repeated alveolar injury. Studies have suggested that microbiota is different between IPF subjects and healthy controls, and that bacterial burden plays a significant role in disease progression.^[3,4]^

Herein, we describe an extremely rare case of tracheal MALT lymphoma associated with IPF.

## 2 Case description

A 73-year-old man who was an ex-smoker (25 pack-years) presented at the outpatient clinic of Yeungnam University Medical Center with a 2-year history of dyspnea on exertion and productive cough, which had worsened 1 month ago. He had IPF, hypertension, and stable angina pectoris and had been treated for nontuberculous mycobacteria 1 year before presenting at the clinic. The patient had no specific family history such as cancer or pulmonary disease. Initial examination revealed crackle at both lung fields. The patient was alert and vital signs were stable.

**Figure 1:**
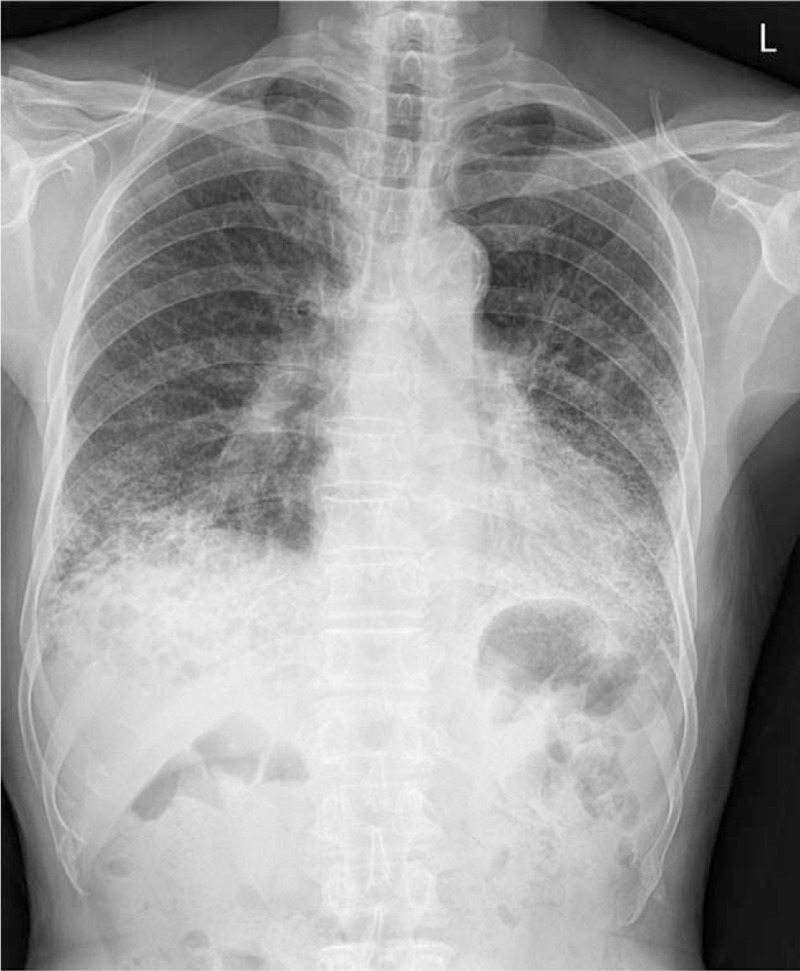
Chest X-ray showed reticulonodular infiltrations at both lung fields.

**Figure 2:**
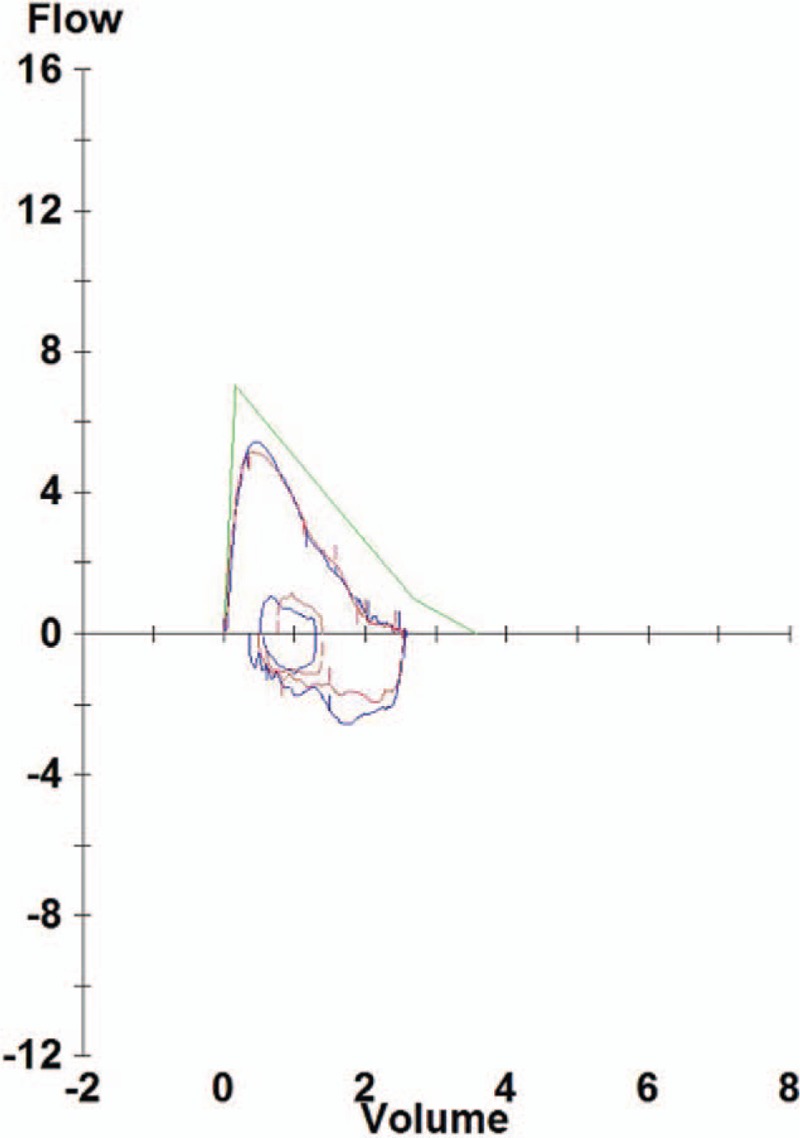
Flow-volume curve suggesting mild restrictive ventilatory defect.

Chest X-ray image revealed reticulonodular infiltrations at both lung fields and associated dense consolidation (Fig. 1). Laboratory examinations revealed decreased hemoglobin (11.6 g/dL) and a 9-fold increase in C-reactive protein (4.37 mg/dL; reference, 0–0.5 mg/dL). Liver and renal function, electrolytes, and lactate dehydrogenase were within normal ranges. Pulmonary function tests revealed a forced vital capacity (FVC) of 2.56 L (71% predicted), forced expiratory volume in the first second (FEV1) of 2.03 L (85% predicted), an FEV1/FVC ratio of 79% (79% predicted), diffusing capacity for carbon monoxide of 11.6 mL/mm Hg/min (74% of predicted), and total lung capacity (TLC) of 3.83 L (71% predicted) (Fig. 2).

**Figure 3:**
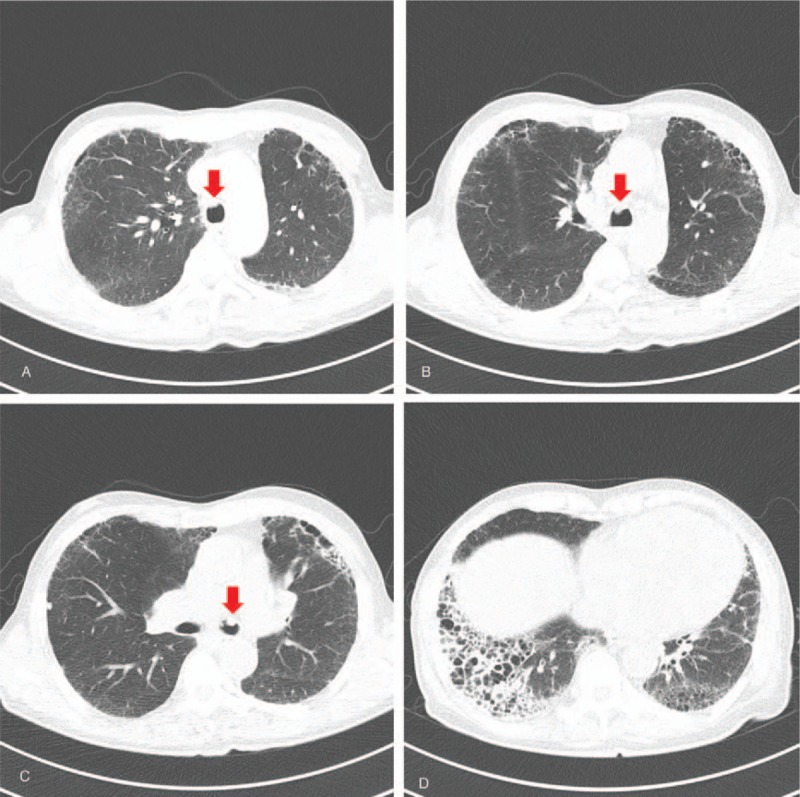
(A–C) Chest CT showed a mass at the distal trachea extending to carina and left main bronchus. (D) Chest CT showed honeycomb change and traction bronchiectasis at peripheral portion of both lower lung fields. CT = computed tomography.

Contrast-enhanced chest computed tomography (CT) revealed a mass at the distal trachea extending to the carina and left main bronchus (Fig. 3A–C). In addition, chest CT also revealed honeycomb changes and traction bronchiectasis at the peripheral portion of both lower lung fields, which suggested usual interstitial pneumonia (Fig. 3D). No evidence of metastatic lymphadenopathy or a lung field mass was observed, and no abnormalities were detected in the great vessels, liver, or adrenal glands.

**Figure 4:**
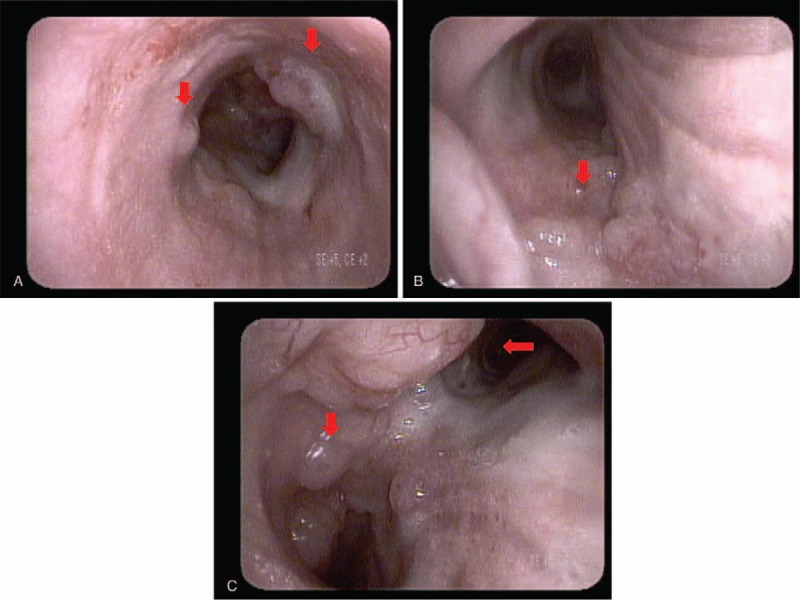
(A–C) Fiberoptic bronchoscopy showed protruding mass at upper and lower trachea extending to carina, proximal right main bronchus, and distal left main bronchus.

**Figure 5:**
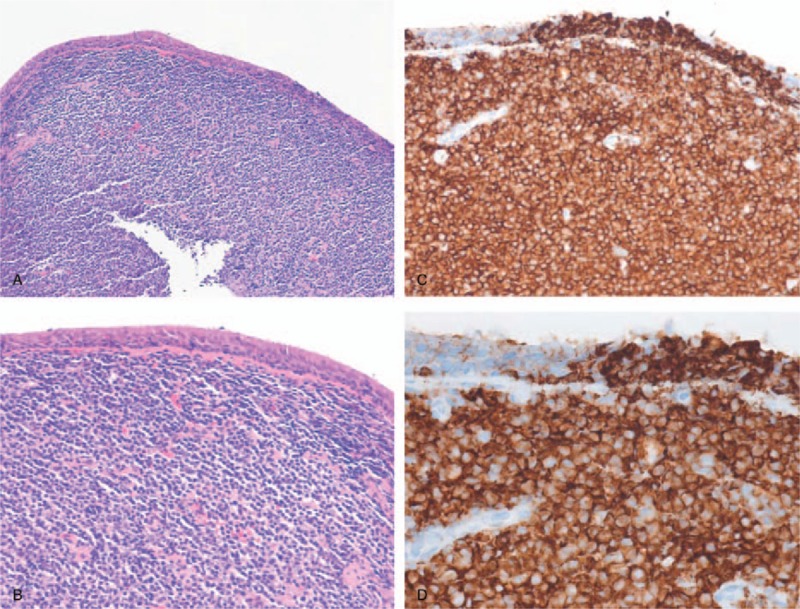
(A and B) Hematoxylin and eosin staining showed atypical small lymphoid tumor cells, diffusely infiltrating the bronchial mucosa, and characteristic lymphoepithelial lesions. (A) Hematoxylin and eosin staining, 100× magnification. (B) Hematoxylin and eosin staining, 200× magnification. (C and D) Immunohistochemical stainings for CD 20 was positive. (C) 200× magnification. (D) 400× magnification.

Fiberoptic bronchoscopy showed a protruding mass at the upper and lower trachea extending to the carina, proximal right main bronchus, and distal left main bronchus (Fig. 4A–C). A biopsy was performed, and subsequent routine hematoxylin and eosin staining revealed atypical small lymphoid tumor cells, diffusely infiltrating the bronchial mucosa, and characteristic lymphoepithelial lesions (Fig. 5A and B). Immunohistochemical (IHC) staining was performed to confirm the diagnosis. Staining for CD 20 was positive, but negative for CD 3, which indicated a B cell origin (Fig. 5C and D). In addition, the Ki-67 labeling index was low (about 15%). Hematoxylin and eosin staining with IHC supported a diagnosis of extranodal marginal zone MALT lymphoma of the trachea.

**Figure 6:**
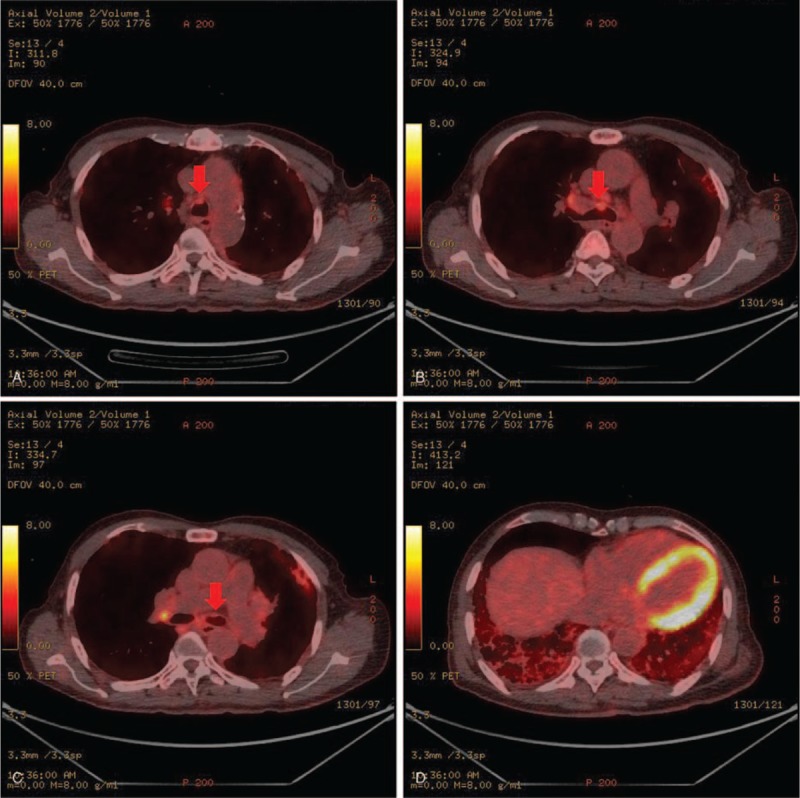
(A–C) Positron emission tomography CT showed FDG avid soft tissue density nodules at trachea and bronchus wall. (D) Diffuse FDG uptake was noted at both lower lung fields. CT = computed tomography, FDG = fluorodeoxyglucose.

Further studies were performed for staging. Positron emission tomography CT revealed fluorodeoxyglucose (FDG)-avid soft tissue density nodules at the tracheal and bronchial wall, suggesting known MALT lymphoma (Fig. 6A–C). Diffuse FDG uptake was noted at both lower lung fields accompanied by interstitial fibrosis with honeycomb changes (Fig. 6D). Gastroduodenal endoscopy revealed linear nodular mucosa at mid antrum, and biopsies revealed chronic gastritis with intestinal metaplasia. Test for helicobacter pylori revealed negative results following Giemsa staining. A colonoscopy revealed no pathologic findings. Bone marrow biopsy was not performed because the patient refused to undergo the procedure. The Ann Arbor stage of the lesion was determined to be IE.

After taking into consideration the age, poor performance status, and comorbidities of the patient and the extent of disease, we utilized an observational approach as a treatment strategy. Currently, the patient is well without any evidence of progression for 12 months since the initial diagnosis.

As this study is a clinical case report, no ethical committee approval was required, which is in compliance with the institutional and national policies concerning research approvals. The family of patient was informed that clinical details and images concerning the case would be submitted for publication, and they provided consent.

## 3 Discussion

Primary tracheal tumors are rare and account for 0.2% of all tumors of the respiratory tract and fewer than 0.1% of all malignant diseases.^[2,5]^ Among them, hematopoietic tumors are rare and principally comprise plasmocytoma and non-Hodgkin lymphoma.^[6]^

Isaacson and Wright first described low-grade extranodal B-cell malignant MALT lymphoma in 1984.^[7]^ Although MALT lymphoma occurs most frequently in the gastrointestinal tract, they have also been described at various nongastrointestinal sites. With regard to nongastrointestinal MALT lymphomas, the lung is one of the most commonly affected organs.^[1]^ Nevertheless, MALT lymphomas affecting the trachea are extremely rare, and most reports in the literature are case reports.^[8–10]^

Only 1 case report in the literature has reported an association of primary tracheal MALT lymphoma with IPF.^[11]^ The association of primary tracheal MALT lymphoma with IPF may be coincidental, but since MALT lymphomas are generally associated with chronic antigenic stimulation in response to cytogenetic abnormalities, autoimmune disease, or chronic pulmonary infection, the association is of particular significance.^[1]^

IPF is the most common form of idiopathic interstitial pneumonia. Although the pathogenesis of IPF is not well understood, an established hypothesis is that it occurs in genetically vulnerable individuals as a consequence of an excessive wound healing reaction following repeated injury to alveolar epithelial cells associated with chronic antigenic stimulation. In addition, studies have suggested that increased bacterial burden predicts disease progression and death in IPF patients and have emphasized significant differences in bacterial load between IPF patients and healthy controls.^[3,4]^ Hence, it is thought that a common predisposing factor, such as chronic antigenic stimulation or cytogenetic abnormalities, may exist for tracheal MALT lymphoma and IPF. Further studies including cytogenetic analysis are needed to identify this relationship.

Clinical manifestation is similar to that of chronic obstructive pulmonary disease or asthma, which often delays the diagnosis. Fiberoptic bronchoscopy and biopsies are essential for diagnosis. Most cases show single or a few localized lesions within the trachea without systemic dissemination.^[9,12–14]^ In the case we report here, MALT lymphoma occurred simultaneously in the trachea and main bronchus. This tracheobronchial involvement is not common among patients with lymphoma.^[8,10,15]^

The prognosis for primary tracheal MALT lymphoma is often favorable. Previous reports have shown the effectiveness of various treatment modalities, including surgical resection, radiation therapy, bronchoscopic therapy, and chemotherapy.^[9,13,16,17]^ Surgical resection is considered if the lesion is localized, and several cases have shown long-term survival after surgical resection.^[9,12,18]^ Radiation therapy also can be considered in localized disease and is shown to be effective, used either alone or in combination.^[9,12]^ Bronchoscopic therapy is an effective method for relieving central airway obstructions caused by tumors. Nd-YAG laser therapy or endoscopic ethanol injection into the tumor shows a good response.^[13]^ Temporary tracheal stenting before chemotherapy and/or radiotherapy is beneficial in severe airway stenosis.^[15]^ The use of chemotherapy is indicated if the lesions are systemic disseminated. Many case reports revealed the effectiveness of chemotherapy including rituximab.^[6,17]^ Rituximab (anti-CD 20 monoclonal antibody) has shown a 70% response rate in MALT lymphoma irrespective of the disease site.^[1]^

Although our patient presented with respiratory symptoms, we selected an observational approach as a treatment strategy. While tumors were identified in the trachea and both main bronchi in this patient, pulmonary function tests revealed restrictive ventilatory defects. Therefore, we concluded that the respiratory symptoms of the patient were caused by underlying IPF rather than tracheal MALT lymphoma. Additionally, due to age and performance status, the patient was not deemed fit enough to endure systemic chemotherapy. Suzuki et al reported that chemotherapy worsened pulmonary fibrosis, causing respiratory failure and death in a patient with primary tracheal MALT lymphoma and IPF. Taking into consideration all of these factors, we decided to utilize an observational approach.

In conclusion, our case shows an association between tracheal MALT lymphoma and IPF, and an observational approach can be a valid treatment option in a patient with other comorbidities and a poor performance status. As there are no randomized clinical trials focusing on tracheal MALT lymphoma, individualized treatment decision is important, and in some cases, simply monitoring the patient might be the most appropriate approach.

## Author contributions

**Conceptualization:** June Hong Ahn, Kwan Ho Lee.

**Resources:** Joon Hyuk Choi.

**Supervision:** Kwan Ho Lee, Jin Hong Chung, Kyeong-Cheol Shin, Eun Young Choi, Hyun Jung Jin.

**Writing – original draft:** June Hong Ahn.

**Writing – review & editing:** Kwan Ho Lee, Jin Hong Chung, Kyeong-Cheol Shin, Eun Young Choi, Hyun Jung Jin.
